# Enhanced Monitoring of Photocatalytic Reactive Oxygen Species: Using Electrochemistry for Rapid Sensing of Hydroxyl Radicals Formed during the Degradation of Coumarin

**DOI:** 10.1021/acs.jpca.3c00741

**Published:** 2023-05-31

**Authors:** Wesley J. McCormick, Clare Rice, Denis McCrudden, Nathan Skillen, Peter K. J. Robertson

**Affiliations:** † The Bryden Centre, Queen’s University Belfast, University Road, Belfast BT7 1NN, U.K.; ‡ Department of Life and Physical Sciences, Atlantic Technological University, Donegal, Letterkenny F92 FC93, Ireland; § School of Chemistry and Chemical Engineering, Queen’s University Belfast, David Keir Building, Stranmillis Road, Belfast BT9 5AG, U.K.

## Abstract

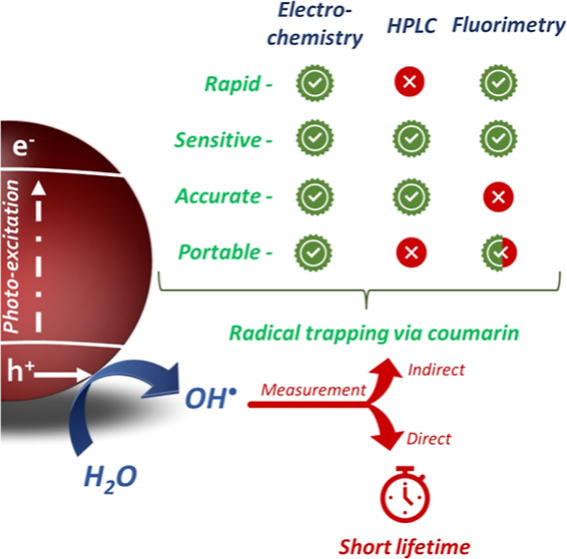

Many recent research studies have reported indirect methods for the detection and quantification of OH radicals generated during photocatalysis. The short lifespan and high reactivity of these radicals make indirect detection using probes such as coumarin a more viable quantification method. Hydroxyl radical production is commonly monitored using fluorescence spectroscopy to determine the concentration of the compound 7-hydroxycoumarin, which is formed from hydroxyl radical attack on coumarin. There are, however, a number of additional hydroxylated coumarins generated during this process, which are less amenable to detection by fluorescence spectroscopy. Consequently, limitations and inaccuracies of this method have previously been reported in the literature. As an alternative approach to those previously reported, this work has developed an electrochemical screening method using coumarin as a OH radical trap, that is capable of in situ monitoring of not only 7-hydroxycoumarin, but all the main mono-hydroxylated products formed. As a result, this technique is a more representative and comprehensive method for the quantification of OH radicals produced by photocatalysts using coumarin as a probe molecule. Moreover, the electroanalytical method provides a portable, rapid, sensitive, and accurate in situ method for the monitoring of OH radical formation without the need for sample preparation.

## 1 Introduction

The application of photocatalysis as a viable approach to environmental remediation^1−4^ and energy production^5−8^ has often been underpinned by the methods used to evaluate catalysts and reactor systems. Materials synthesis continues to be the primary area of research in this field, which is evident from the extensive range of literature reporting novel catalysts for numerous applications,^9,10^ often with the objective of operating under visible irradiation.^11,12^ As a result, there has always been a certain amount of focus on how these catalysts are evaluated to determine their level of activity.^13,14^ In relation to pollutant removal, this often involves the use of chemical probes or model compounds, which are a simplified representation of real-world contaminants. The purpose of probes and model compounds is primarily to assess how active a material is, which is then often displayed using metrics such as photonic efficiency or an apparent quantum yield. Probes can also provide an insight at a fundamental level by exploring the physical chemistry of the photocatalytic process, specifically, monitoring the generation of reactive oxygen species (ROS). ROS include hydroxyl radicals (OH^•^), superoxide anions (O_2_^•–^), and H_2_O_2_, with OH^•^ often playing a major role in oxidation pathways due to having a significantly oxidizing redox potential of 2.8 V vs NHE. A number of excellent and informative papers in the literature have covered the generation, detection, and impact of these radical species.^15−17^

In recent years, coumarin has been frequently reported as a probe molecule for assessing photocatalysts based on quantifying OH radical formation.^18−22^ As a probe molecule, coumarin has several advantages including cost effectiveness, reproducibility, and ease of monitoring using spectroscopic techniques. Upon irradiation of suspended TiO_2_ in a solution of coumarin, multiple hydroxylated products can be formed based on the site of OH radical attack. 7-Hydroxycoumarin (7-OHC) is, however, the only hydroxylated product with the ability to produce a strong fluorescent signal, making its quantification relatively simple by fluorometric analysis. Furthermore, fluorometric analysis has been shown to be a sensitive method for the detection of 7-OHC with previous reports demonstrating analysis with standards at 5 nM (0.81 μg/L).^23^ As this is an indirect method of radical quantification, in that product, concentration is measured as a result of OH^•^ attack, the ratio of products formed must also be considered. A study which used coumarin to trap OH^•^ radicals generated by γ-ray irradiation determined that 6.1% of products formed were 7-hydroxycoumarin.^23^ This value has subsequently also been adopted in photocatalytic studies using coumarin as an indirect method for determining yields of OH^•^.^24−26^

Despite the favorable properties of coumarin as an approach to quantitatively assess photocatalytic activity by the measurement of OH radical production, limitations of its application have also been reported in the literature. In 2019, Leandri et al.^18^ explored the use of coumarin as a probe for TiO_2_ photocatalysis with a view toward determining the suitability of the compound for future work. Following a thorough investigation, the authors concluded that coumarin “cannot be used to probe the maximum photocatalytic efficiency of TiO_2_ or any other solid photocatalyst.”^18^ This conclusion was based on the limited solubility of coumarin in water coupled with its low affinity for oxide surfaces. In addition, an inner filtering effect was also reported by Leandri and coworkers, where coumarin absorbed a portion of the light required to excite the 7-hydroxycoumarin leading to inaccurate determination of the product at higher coumarin concentrations.^18^ The authors, however, demonstrated that this could be overcome using a correction factor. Subsequent debate in the literature described this correction as complicated and that the problem of inner filtering can be more efficiently overcome by diluting the sample prior to fluorescence measurements.^27,28^ While these findings are interesting, they do unequivocally highlight the need to ensure a high degree of scrutiny is employed when using chemical probes for photocatalysis. Methods of monitoring photocatalytic processes with chemical probes are underpinned by their ability to provide accurate assessment of the reaction system, ideally with minimal time and expertise required. As such, the reliance on these routinely used spectroscopic methods is often high, which further emphasizes the need for robust testing protocols. Furthermore, in the example of coumarin, that reliance is almost entirely placed on the concentration of 7-hydroxycoumarin generated, which is calculated from a percentage reported for a homogeneous radiation chemical process and not heterogeneous photocatalysis. This key point has only briefly been addressed in recent literature; however more importantly, there are no examples exploring how to determine an accurate and relative proportion of 7-OHC as a product of the photocatalytic degradation.

Therefore, it is crucial to consider the probe reaction with respect to all products formed to ensure an accurate evaluation of the photocatalyst and its ability to generate OH radicals. The use of high-performance liquid chromatography (HPLC) analysis to monitor the decrease in coumarin concentration and the formation of hydroxylated products during photocatalytic reactions has been limited to relatively few studies.^19,29,30^ Provided that appropriate standards are available, more inclusive information could be obtained with regard to all the mono-hydroxylated (m-OHC) products formed. Compared to the fluorescence measurements of only 7-OHC, this would be a valuable alternative approach. Although an established and highly accurate method, a significant disadvantage of HPLC is the substantial times required for elution of target analytes, which renders it unsuitable for rapid analysis. In addition, HPLC is unable to provide real-time or in situ analysis of reactions, which is highly desirable given the kinetics often associated with photocatalysis.

In contrast to HPLC, electrochemical techniques can deliver a rapid analysis of compounds with the added benefit of being cost effective and suitable for portable and in situ operation. The use of electrochemical detection coupled with photocatalytic systems has previously been reported for monitoring the degradation of pesticides, industrial pollutants, and pharmaceutical contaminants.^31−34^ To date, however, the use of such a method for monitoring coumarin degradation and subsequent generation of hydroxycoumarin via photocatalytic reactions has never been explored.

Previous studies that involved electrochemical detection of coumarin focused on monitoring coumarin levels in foodstuffs, plants, and oils as it is a physiologically toxic substance at certain levels in the body.^35−37^ The electroanalytical reduction of coumarin has been shown in these studies to occur between −1.4 and −1.6 V vs Ag/AgCl reference electrode, depending on the pH of the electrolyte or buffer used for the analysis. Moreover, all mono-hydroxylated coumarins, which may possibly be formed, are also known to be electrochemically active. These molecules are irreversibly oxidized at potentials ranging from 0.3 to 1.1 V vs Ag/AgCl reference electrode, with the concentrations of products formed determined from the peak areas of corresponding standards.^38^

While coumarin has limitations and may be unable to probe the maximum photocatalytic efficiency of a catalyst, it also has advantages, providing more substantial analysis can be deployed. Therefore, presented in this work is the development of a novel electrochemical detection method for monitoring the quantity of OH radicals produced during photocatalytic reactions when using coumarin as a probe. It was found that both qualitative and quantitative analysis could be provided using this rapid detection method. The results of the electrochemical study were also correlated using HPLC analysis to confirm the validity and accuracy of the technique. Furthermore, the deployed method allowed in situ analysis of the reaction products formed during the photocatalytic process. This key characteristic demonstrates the potential of the technique for providing a more representative and comprehensive method for assessing photocatalytic activity while overcoming previously reported limitations.

## 2 Experimental Methods

### 2.1 Chemical Reagents

Coumarin (99%), 7-hydroxycoumarin (99%), 6-hydroxycoumarin (96%), 4-hydroxycoumarin, 3-hydroxycoumarin (98%), and potassium phosphate solutions (1.0 M) were purchased from Sigma-Aldrich, USA. 8-Hydroxycoumarin was obtained from MedChemExpress. 5-Hydroxycoumarin was synthesized with slight modification according to a method by Adams and Bockstahler (1952).^39^ The Aeroxide P25 photocatalyst was purchased from Evonik Degussa, Germany. Methanol (≥99.9%), acetonitrile (≥99.9%) formic acid, and acetic acid (≥99.8%) were of HPLC grade and purchased from Sigma-Aldrich. Millipore water (18 MΩ cm) was used for the preparation of all aqueous solutions. All chemicals used were of analytical reagent grade and used as received.

### 2.2 Photocatalytic Reactor Setup

The photocatalytic experimental setup comprised a borosilicate glass beaker containing 100 mL of phosphate buffer with differing concentrations of coumarin (100–1000 μM) and 50 mg of photocatalyst. A magnetic stir bar was used to agitate the solution at a constant rpm. The irradiation array consisted of a UV-LED strip (SMD3528-600) of 30 light-emitting diodes (LEDs) with a peak wavelength of 365–370 nm and at a beam angle of 120°. The LED strip was mounted on the inside of a cylindrical wire mesh support. The LED light strip was powered by an AC–DC power supply (MPW Ea1050A-120) with an output of 12 V and 5 A. The reaction beaker was placed on a stirring plate inside the wire mesh mount as shown in Figure S1A. The distance between the light source and beaker was approximately 2 cm. The reaction solution was stirred in the dark to allow a state of equilibrium to be reached before irradiation was switched on and samples (in duplicate) were taken at regular intervals for analysis. Samples (1 mL) were removed from the reaction vessel at selected time intervals, with one sample centrifuged for 10 min at 3000 rpm and used for HPLC analysis and the duplicate subjected to electrochemical analysis. For comparison with the spectroscopic technique, triplicate samples were removed from the reaction vessel at selected times. Control experiments were performed in the absence of light (dark control) and catalyst (photolysis/light control).

### 2.3 Fluorescence Spectroscopic Analysis of 7-OHC

Emission spectra was recorded using a PerkinElmer LS-55 Fluorescence Spectrometer with the excitation wavelength of 332 nm and emission wavelength of 456 nm. Both the adsorption and emission spectra were recorded using quartz Suprasil cuvettes. Quantification of 7-OHC was performed by a five-point external calibration method.

### 2.4 HPLC Analysis

Concentrations of coumarin and its hydroxylated derivatives were determined using HPLC (Shimadzu LC-2010HT). The analytes were separated using a Spherisorb ODS (2) column (250 mm × 4.6 mm i.d., 10 μm) (Phenomenex, Torrance, CA). The mobile phase consisted of 5% formic acid (A) and methanol/acetonitrile (80:20, v/v) (B). Separation was achieved by gradient elution using the following program: 0–15 min (5–27% B); 16–27 min (27% B); 28–30 min (27–5% B); and 30–60 min (5% B). The column temperature was set to 30 °C, the flow rate was 1.0 mL/min, and a sample size of 25 μL was injected in each analysis. Data were acquired at 271 nm. Quantification of coumarin, 3-, 4-, 5-, 6-, 7-, and 8-OHC and 6,7-diOHC was performed by a five-point external standard calibration method.

### 2.5 Electrochemical Analysis

Electrochemical analysis was performed using a hand-held potentiostat (CHI 1220C, CH Instruments, USA) and a personal computer for data storage and processing. A three-electrode configuration was used throughout with a bare glassy carbon electrode (*Ø* 3 mm) as the working electrode, an Ag/AgCl (3 M KCl) reference electrode, and a platinum wire counter electrode (Figure S1B). All measurements were performed in phosphate buffer (pH 7.4) at room temperature (20 ± 3 °C) in a 2 mL cell. Square-wave voltammetry (SWV) was used to monitor the degradation of coumarin in the potential window of −1.4 to −1.7 V. Likewise, SWV was also used for the detection and monitoring of the hydroxylated coumarin derivatives from 0.3 to 1.0 V. Quantification of probe and its derivatives was performed by a five-point external standard calibration method.

## 3 Results and Discussion

### 3.1 Fluorescence Spectroscopic Analysis

**Figure 1:**
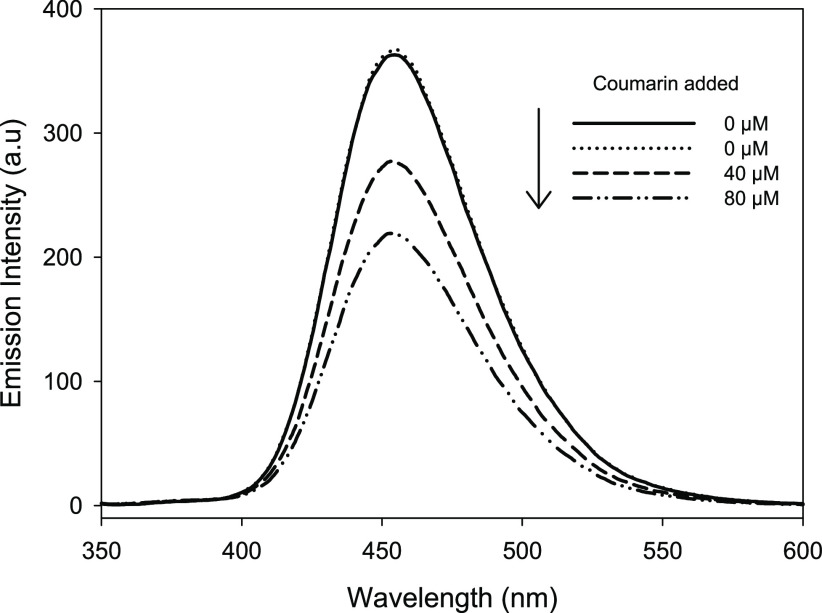
Fluorescence signal recorded for 2 μM 7-OHC and after coumarin additions.

As previously stated in the literature, coumarin may cause an inner filtering effect influencing the effectiveness of fluorescence spectroscopy for detection of 7-OHC. To validate this observation within this study, an initial investigation was undertaken to determine the potential extent of this effect. As expected, the intensity of the fluorescence signal for a 2 μM 7-OHC solution was shown to decrease with increasing concentration of coumarin (Figure 1). This decrease is clearly due to competing absorption of the fluorescence light between coumarin and 7-OHC. As a result, previous reports using fluorescence monitoring may have underestimated the concentration of 7-OHC produced.^22,40,41^ Therefore, the predicted value of hydroxyl radical generation on the photocatalyst surface would in turn have been lower than the true concentration. While this confirms the findings reported in the literature, it also further emphasizes the requirement for a nonspectroscopic method. It is evident that the accurate determination of hydroxyl radical generation on photocatalyst materials requires a method, which is not subjected to the aforementioned interferences.

### 3.2 HPLC Analysis of Coumarin Probe System

**Figure 2:**
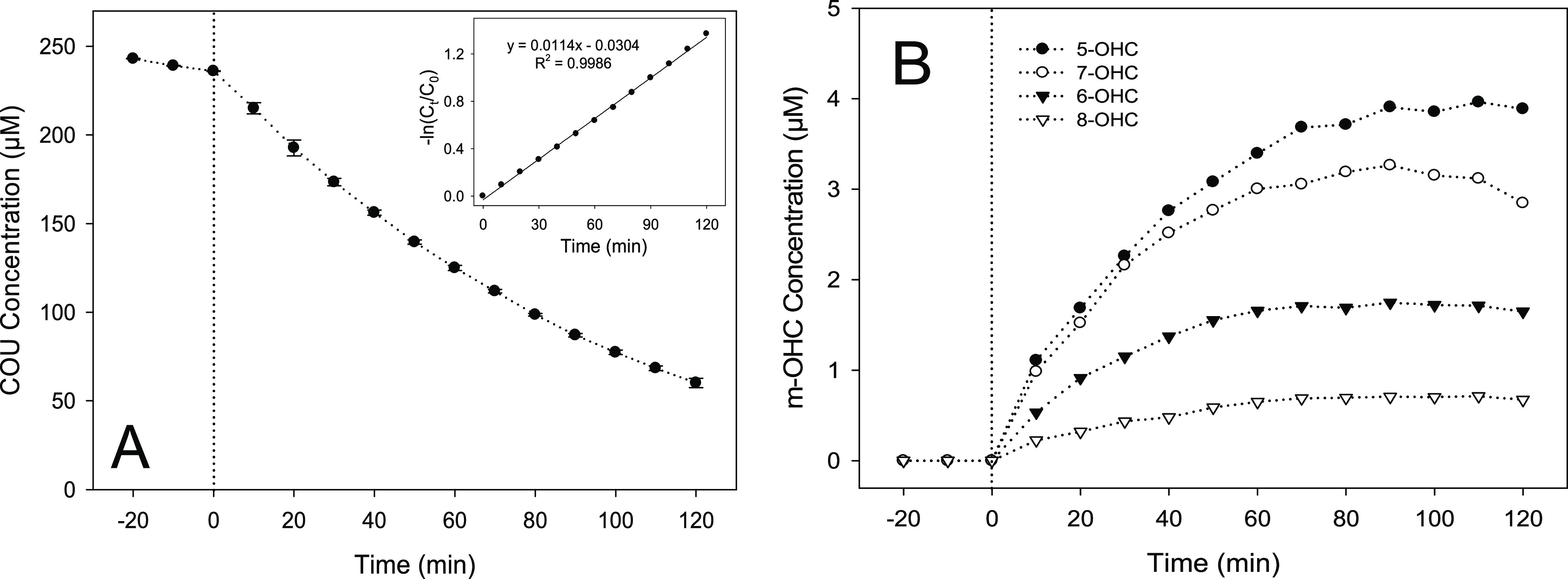
Concentration vs time plots displaying the degradation of coumarin (A) and formation of the four main hydroxylated products (B) using 250 μM starting coumarin concentration. In both, time 0 on the *x*-axis represents the point at which irradiation was started. Insert in (A) shows a plot of −ln(*C*_*t*_/*C*_0_) vs time to confirm first order kinetics.

HPLC was used to generate a baseline data set, which provided confirmatory analysis for the electrochemical method developed (Figure S2). To demonstrate the degradation profiles observed for photocatalytic coumarin oxidation, Figure 2A presents data for a starting concentration of 250 μM while other coumarin concentrations are displayed in Figure S3. In addition, Figure 2B shows the time–concentration profiles of the mono-hydroxylated products formed during this reaction. M-OHC profiles for starting concentrations of 100 and 500 μM coumarin in the photocatalytic reaction are shown in Figure S4.

**Figure 3:**
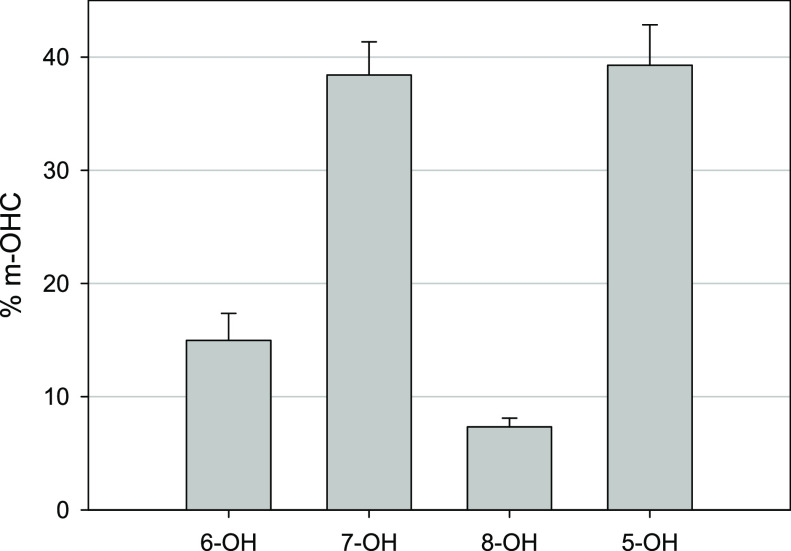
The average % ratio contribution of the m-OCH formed over all concentrations for all times.

As shown in the data, 5-OHC was found to be the most abundantly formed hydroxylated coumarin with a maximum yield of 4.0 μM following 110 min of irradiation. This was followed by 7-OHC (3.3 μM), 6-OHC (1.8 μM), and 8-OHC (0.7 μM), which reached maximum yields after 90-min irradiation. In addition, no peaks were observed for 3-OHC and 4-OHC. This is in good agreement with Louit et al.^30^ for the detection of hydroxyl radicals using coumarin as a probe during gamma radiolysis although in this study, 4-OHC was found to be more abundant than 8-OHC and 3-OHC was present at very low levels. A separate study using HPLC^29^ also confirmed the absence of 3- and 4-OHC and the presence of 6- and 7-OHC by co-elution with appropriate standards, while two remaining peaks were tentatively identified as 5-OHC or 8-OHC. As part of the same study, 7-OHC was reported to account for approximately 24% of all 4 mono-hydroxycoumarins formed during the photocatalytic reaction when analyzed separately by GC-FID. In this study, it was found that 7-OHC accounted for approximately 37% of all mono-hydroxycoumarins. The percentage ratio for all m-OCHs formed was determined and is summarized in Figure 3 (as an average), with further supporting data for individual concentrations shown in Figure S5. The results demonstrated the ratio of m-OCH remained relatively constant over different probe concentrations at selected time intervals of 30, 60, 90, and 120 min (Figure S5). Furthermore, the standard deviation shown in Figure S5 suggests that the average contribution remained fairly constant over different concentrations and at different times during the photocatalytic reaction.

An interesting observation from the confirmatory HPLC analysis was the overall degradation profiles and product distribution for coumarin and m-OCH at different starting concentrations. It was noted that at a starting concentration of 100 μM coumarin, all m-OCH reached a peak yield after approximately 60 min before then undergoing subsequent degradation. In contrast, at concentrations of 250 and 500 μM, all m-OHC reached close to steady-state production, with the notable exception of 7-OHC from 250 μM coumarin. Furthermore, a greater degree of variation in % m-OHC at different time points for 100 μM coumarin was also observed (Figure S5A). Both of these observations suggest that it is crucial to select an appropriate starting concentration for monitoring OH radical generation via photocatalytic degradation of coumarin. In view of this, it is desirable to consider higher starting concentrations of coumarin that facilitate steady-state production of the target hydroxycoumarins. While a rapid reaction rate that achieves a high conversion is often beneficial for photocatalytic applications, it is not optimal for the system described in this study. The exhaustion of the substrate (coumarin) and subsequent oxidation of the products (hydroxycoumarins) means that the photocatalytic efficiency is not easily determined. A sensitive and accurate monitoring method must be deployed to measure the maximum yield of 7-OHC without the risk of further oxidation from ROS. Based on the data presented in this study, the authors recommend a coumarin concentration of ≥500 μm to ensure the generation of hydroxycoumarins reach steady state and prevent subsequent oxidation. This is evident from Figure S4, which shows in the case of 6- and 8-OHC, steady-state production was achieved after 120 min of irradiation.

### 3.3 Electrochemical Analysis of Coumarin Probe

**Figure 4:**
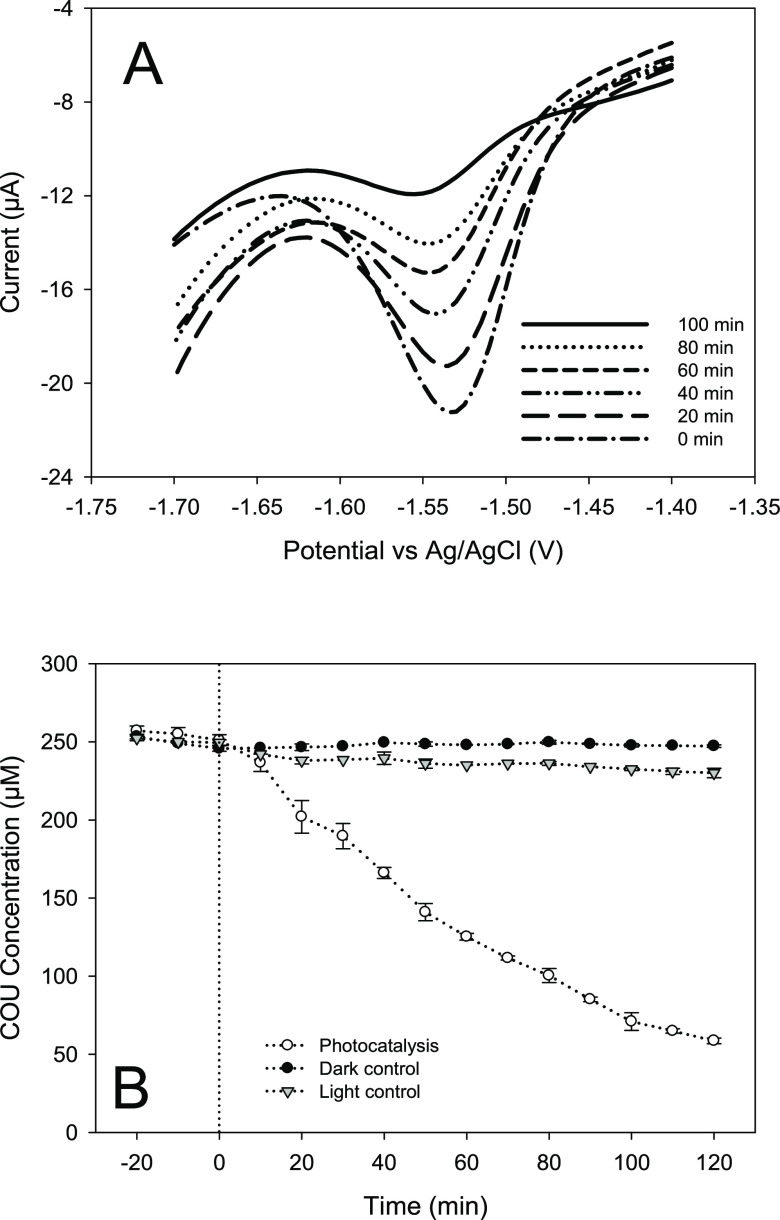
Square-wave voltammograms for a 250 μM coumarin solution at selected time intervals during irradiation in the presence of TiO_2_ (A). Time–concentration profile of coumarin under photocatalytic, photolytic (light control), and catalyst only conditions (dark control) (B). (*n* = 3).

While the data generated from the HPLC analysis allowed the ratio of products formed to be determined, its use as a screening method was limited due to the time constraints associated with its operation and sample preparation procedures. The electrochemical method developed in this study was capable of overcoming this issues as a result of coumarin and all the m-OHCs, which may be formed during photocatalysis, being electrochemically active.^38^Figure 4 displays the photocatalytic degradation of coumarin monitored via the electrochemical method, with the coumarin reduction peak area at ∼−1.54 V shown to be decreasing with irradiation time (Figure 4A). The level of degradation analyzed and shown in Figure 4B corresponds to that recorded by HPLC analysis and shown in the previous figure; overall removal rates of 1.48 and 1.59 μM min^–1^ were determined from HPLC and electrochemical analysis, respectively. In addition, and as expected, the control experiments confirmed that photocatalysis was primarily responsible for the decrease in coumarin concentration due to hydroxylation (Figure 4B). Under photolytic (UV only) conditions, no significant degradation was observed, while in the absence light, the dark control displayed minimal coumarin degradation, which also highlights coumarins’ low affinity for oxide-based photocatalysts such as TiO_2_.

**Figure 5:**
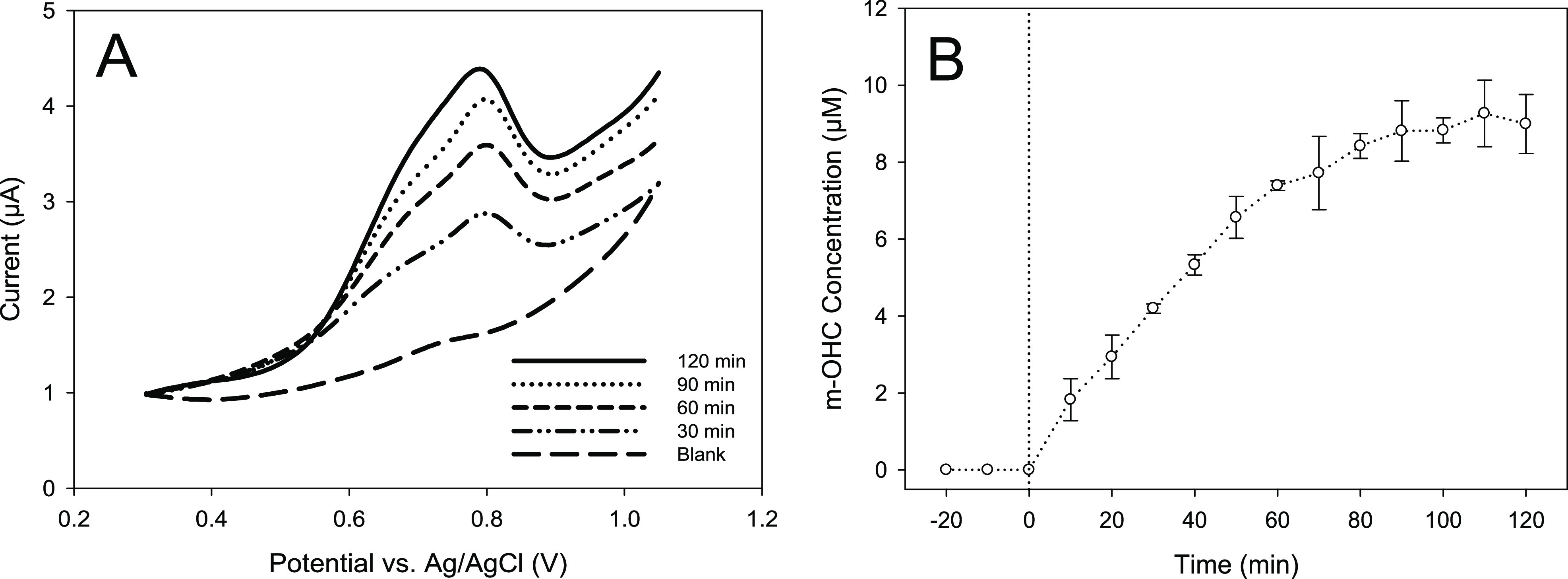
Square-wave voltammograms at selected time intervals for m-OCH produced from a starting probe concentration of 250 μM coumarin (A). Concentration of m-OCH produced with irradiation time (B).

Although each of the m-OHCs standards have a specific electrode potential, of which 3- and 4-OHC are sufficiently separated, electrode potentials for 5-, 6-, 7-, and 8-OHC are relatively close and complete separation of the individual peaks was not achievable. Figure 5 displays that voltammograms of aliquots removed from the reaction vessel at various time intervals when the coumarin starting concentration was 250 μM. This demonstrates that no peaks were evident for 3- or 4-OHC, which was also seen when conducting HPLC analysis. The area of the peak from approximately 0.5–0.9 V, however, was considered to be representative of the cumulative amount of 5-, 6-, 7-, and 8-OHC produced as the peak potentials were within the electrode potential range of the individual m-OCH formed.

Using the ratios determined from the HPLC analysis in conjunction with calibration standards generated from electrochemical analysis allowed the concentration of each individual m-OHC to be determined from the total area of the voltammetric peak. Calibration data were collated for coumarin and the mono-hydroxylated derivatives for each analytical technique (Table S1).

### 3.4 Comparison of Methods for Monitoring the Detection and Quantification of Hydroxycoumarins

**Figure 6:**
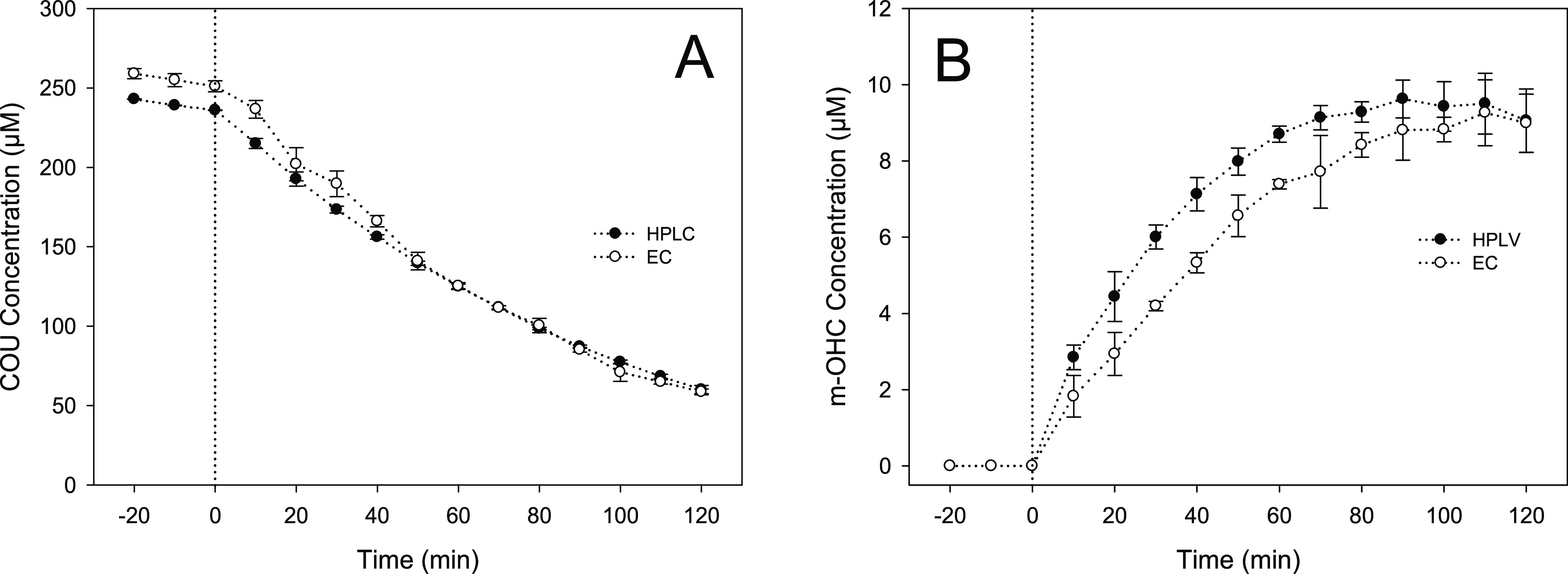
Comparison of HPLC and electrochemical analysis for the determination of the total concentration for all m-OHC formed with irradiation time for 250 μM coumarin.

The rate of photocatalytic coumarin degradation monitored using HPLC and electrochemical methods were in excellent agreement using the 250 μM coumarin concentration (Figure 6A). This was further supported by the comparison between other coumarin concentrations as shown in Figures S6–S8. Summation of individual m-OCHs allowed the total concentration of the formed m-OCHs to be accurately calculated, which was then compared to HPLC results for different probe concentrations. Figure 6B shows the formation of the mono-hydroxycoumarins when monitored by both HPLC and electrochemistry using a starting coumarin concentration of 250 μM (with additional starting concentrations shown in Figure S7). The similarity between the profiles confirmed that the electrochemical analysis was in good agreement with the established HPLC analytical method. A key advantage of the electrochemical method, however, was its ability to generate the data via a portable and rapid in situ sensing system which required no sample preparation. Most significantly, there was no need to separate the photocatalyst from the solution prior to analysis, which facilitated real time in situ monitoring of the photocatalytic process.

**Figure 7:**
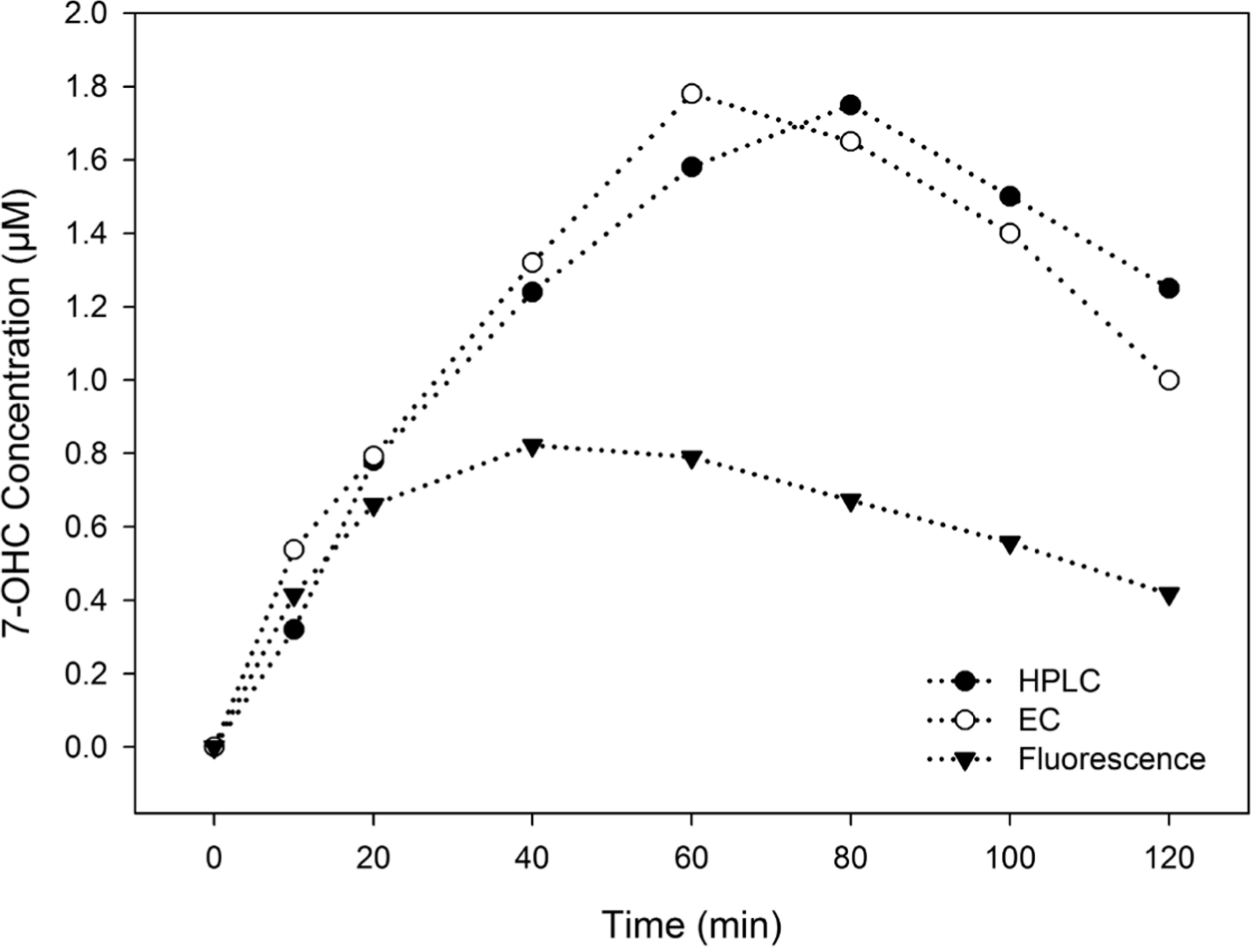
Concentration with irradiation time profiles for 7-OHC monitored by HPLC, electrochemistry, and fluorescence spectroscopy. Note that, the fluorescence signal shown in this figure is uncorrected. The reaction mix contained 100 μM coumarin and 0.5 g/L TiO_2_.

The formation of 7-OHC during a 2-h photocatalytic reaction was monitored and compared using the electrochemical method, HPLC, and the more routinely reported method of fluorescence spectroscopy (Figure 7). For this particular set of experiments, a starting concentration of 100 μM was selected to highlight key observations when carrying out such a study. As previously mentioned, at a starting concentration of 100 μM, the oxidation of 7-OHC begins at 40–80 min of irradiation. As a result, this prevents a total OH production rate or yield from being obtained and ultimately reduces the accuracy of the calculated OH radical concentration. This is further complicated by the nonselective nature of ROS attack and the multiple reactions, which occur during the photocatalytic process. While the efficiency of a photocatalytic reaction is often determined by calculating the initial rate, the maximum yield of 7-hydroxycoumarin in this reaction is desirable as an indication of a photocatalysts ability to produce a powerful oxidant. The more significant observation, however, is the clear variation between the calculated concentrations using fluorescence spectroscopy compared to HPLC and the electrochemical method. It is evident from the data that the spectroscopic approach provides an inaccurate determination of 7-OHC (∼0.8 μM) and subsequently a poor evaluation of OH radicals generated. This corresponds and validates the limitations previously mentioned in the literature,^18^ which highlighted the issue associated with an inner filtering effect when conducting fluorescence analysis. In contrast, the HPLC analysis and electrochemical method were not restricted by such limitations and subsequently determined higher yields of 7-OHC (∼1.8 μM), which are expected to be a more accurate estimation of radical concentration. Moreover, the electrochemical method was the only approach that allowed that determination to occur via in situ monitoring and without the need for catalyst separation.

## 4 Conclusion

Accurate monitoring of OH radicals produced during photocatalytic reactions is of great importance when accessing the efficiency of individual photocatalysts. Indirect measurements, when using coumarin as a probe, have previously been reported by the determination of the fluorescent 7-OHC produced by the photocatalytic degradation of coumarin. While this provides a rapid screening method with good sensitivity, it has limitations due to adsorption of the excitation light by the probe and the need for filtration before analysis. It also is limited to the detection of only one of several mono-hydroxylated coumarin products that may be generated as part of the photocatalytic process. In this study, HPLC and an electrochemical method were deployed to provide a more accurate, sensitive, and robust approach to monitoring the photocatalytic degradation of coumarin. HPLC analysis allowed each mono-hydroxycoumarin produced to be quantified and their ratio was shown to remain relatively constant at different times over various concentrations. Overall percentage values found for 5-, 6-, 7-, and 8-OHC were 39 ± 3.5, 15 ± 2.4, 38.5 ± 2.9, and 7.5 ± 0.8, respectively. To the best of our knowledge, this is the first time that all 6 mono-hydroxycoumarin standards have been used in conjunction with HPLC separation to allow the accurate determination of the total concentration of the mono-hydroxylated products formed with time over a range of concentrations. Using these ratios, electrochemical analysis allowed the quantification of all mono-hydroxycoumarins produced in the reaction vessel. HPLC and electrochemical detection were in good agreement for the quantification of 7-OHC compared to the much lower values being recorded for the more common fluorescence method, which was attributed to the inner filtering effect. Coumarin, as a chemical probe, can be monitored by the electrochemical technique of SWV and can provide greater efficiency in assessing OH radical formation from photocatalysts by circumventing some of the previously cited limitations when using this probe. These include avoiding potential sources of errors associated with the requirement for sample dilution before fluorescence measurements or the need for complex corrections at higher probe concentrations. As an alternative, HPLC analysis can provide more accurate monitoring of the reaction and also has limitation such as the need for costly instruments and complex sample preparation. A favorable alternative to both spectroscopy and HPLC is the electrochemical method presented in this paper, which requires no sample preparation or filtration and inexpensive instrumentation, and allows for a rapid generation of results via an in situ portable technique.

## Supporting Information Available

Data and findings presented in this manuscript are supported by the additional work shown in the accompanying file which includes the following: images of the reactor experimental setup, chromatogram detailing the detection of hydroxycoumarins, time–concentration profile graphs for the photocatalytic degradation of coumarin at different starting concentrations, a summary table of the data obtained from different analytes using HPLC, electrochemistry and fluorescence, data on the ratio of hydroxycoumarins produced during the photocatalytic degradation of coumarin, and time–concentration profile graphs for the comparison of HPLC and electrochemistry for monitoring the photocatalytic degradation of coumarin and formation of hydroxycoumarins (PDF)
