# Supplementary material for: Enhanced Monitoring of Photocatalytic Reactive Oxygen Species: Using Electrochemistry for Rapid Sensing of Hydroxyl Radicals Formed during the Degradation of Coumarin
Source: J Phys Chem A. 2023 May 31;127(23):5039–47. doi: 10.1021/acs.jpca.3c00741 (PMC10278141; doi:10.1021/acs.jpca.3c00741)
Supplement: Supplementary file 1 — jp3c00741_si_001.pdf [file jp3c00741_si_001.pdf]

Supporting Information for Publication

for

Enhanced Monitoring of Photocatalytic Reactive Oxygen Species: Using Electrochemistry for Rapid Sensing of Hydroxyl Radicals Formed During the Degradation of Coumarin.

Wesley J. McCormick [1,2,3], Clare Rice [1,3], Denis McCrudden [1,2], Nathan Skillen [1,3], Peter K. J. Robertson [1,3]

1. The Bryden Centre, Queen's University Belfast, University Road, Belfast, BT7 1NN, Northern Ireland, United Kingdom.
2. Department of Life and Physical Sciences, Atlantic Technological University Donegal, Letterkenny, F92 FC93, Ireland.
3. School of Chemistry and Chemical Engineering, Queen’s University Belfast, David Keir Building, Stranmillis Road, Belfast BT9 5AG, Northern Ireland, United Kingdom.

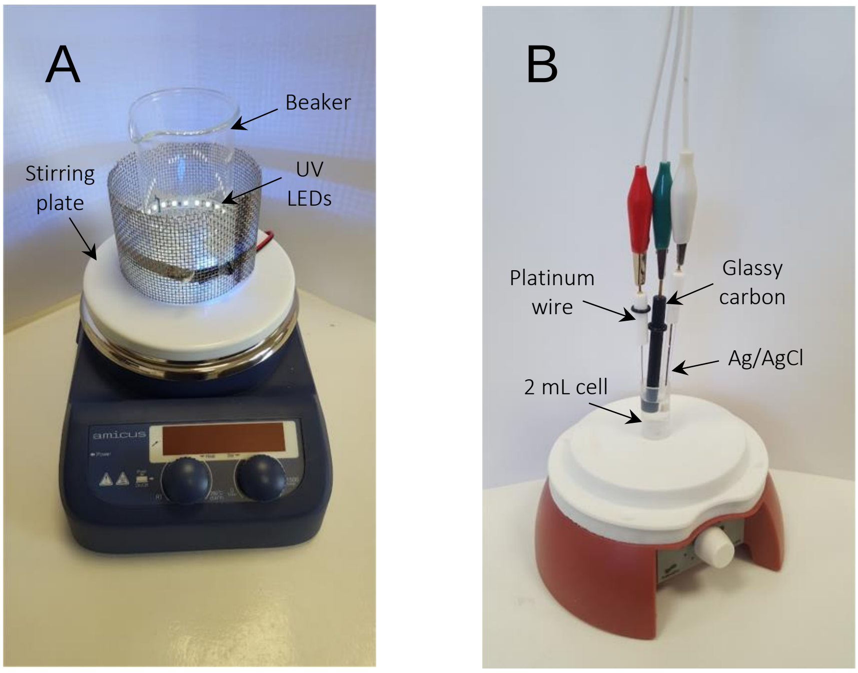

Figure S1. Set-up for photocatalytic experiments (A) and three-electrode configuration consisting of a glassy carbon as a working electrode, Ag/AgCl electrode as a reference electrode and a platinum wire as a counter electrode. Also shown is the 2 mL cell with electrodes inserted in sample (B).

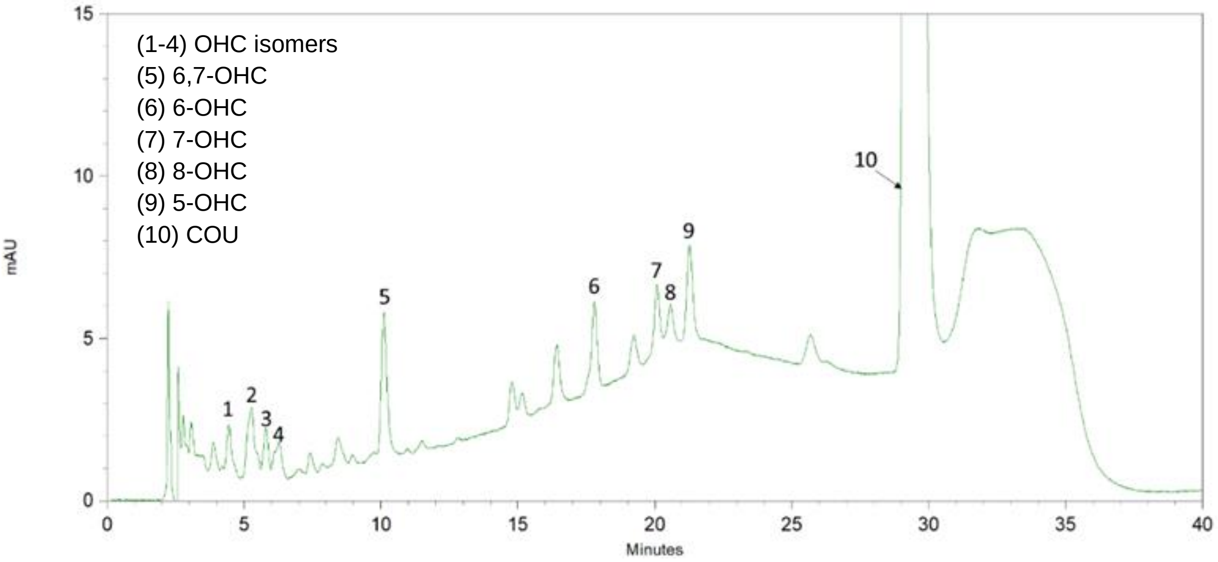

Figure S2. HPLC chromatogram of a 250 µM coumarin irradiated solution after 120 minutes. Detection was at 273nm.

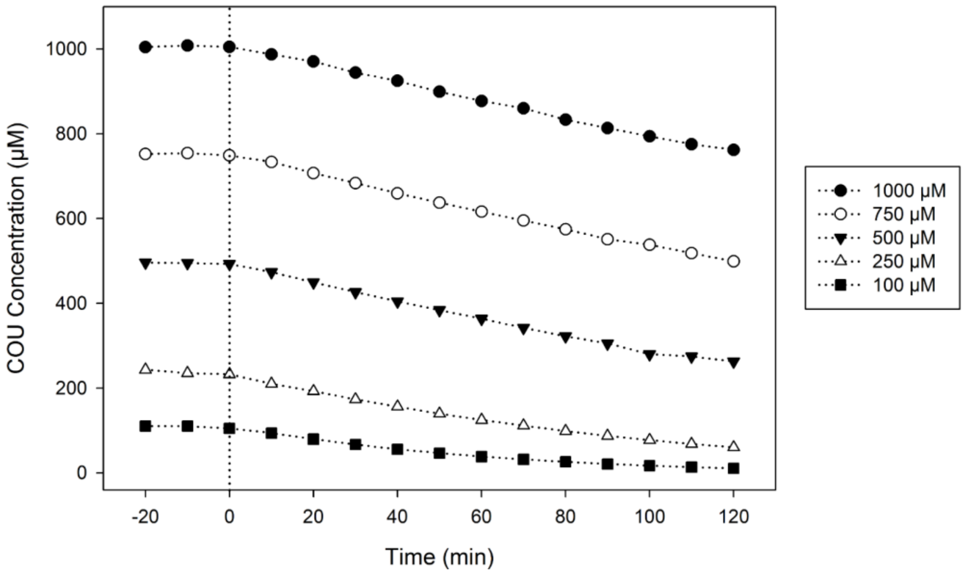

Figure S3. Photocatalytic degradation of coumarin with irradiation time monitored by HPLC when using different concentrations of coumarin.

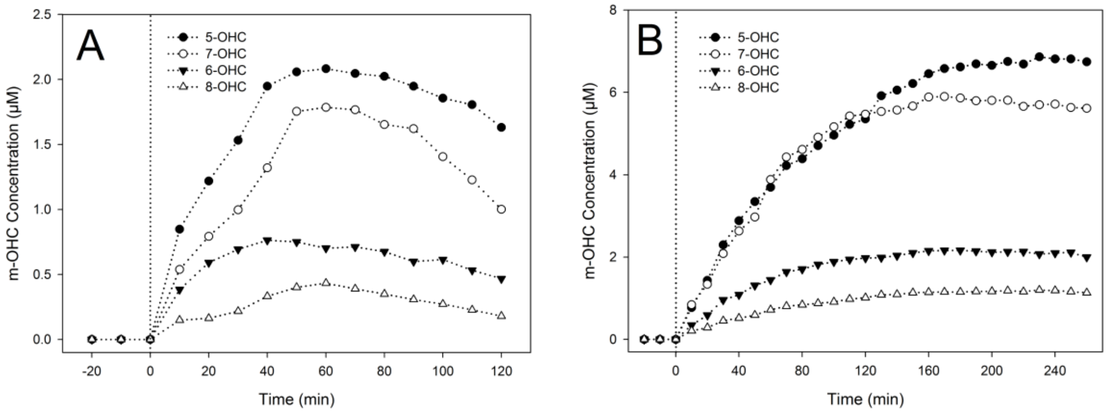

Figure S4. Concentration vs. time plots displaying the formation of the four main hydroxylated products using 100 µM (A) and 500 µM (B) starting concentrations of coumarin.

Table S1: Data obtained for different analytes using the various analytical methods.

**Technique**	**Analyte**	**Concentration (μM)**	**Linear Regression Equation**	**R^2 value**	**Retention time (min)**	**Potential (V)**	**Wavelength (Emission Intensity) (nm)**
HPLC	Coumarin	100 - 1000	$y=10967x$	0.9993	29.30	-	-
	5-OHC	1 - 10	$y=5139.1x$	0.9963	21.27	-	-
	6-OHC	1 - 5	$y=8795.6x$	0.9991	17.78	-	-
	7-OHC	1 - 10	$y=2729.7x$	0.9996	20.08	-	-
	8-OHC	0.5 - 2.5	$y=6868.7x$	0.9981	20.57	-	-
EC	Coumarin	100 - 1000	$y=0.0041x$	0.9997	-	-1.54	-
	5-OHC	1 - 10	$y=0.3505x$	0.9952	-	0.76	-
	6-OHC	1 - 5	$y=0.2143x$	0.9995	-	0.65	-
	7-OHC	1 - 10	$y=0.1712x$	0.9987	-	0.73	-
	8-OHC	0.5 - 2.5	$y=0.2816x$	0.9968	-	0.72	-
Fluorescence	7-OHC	2 - 10	$y=195.45x$	0.9972	-	-	455

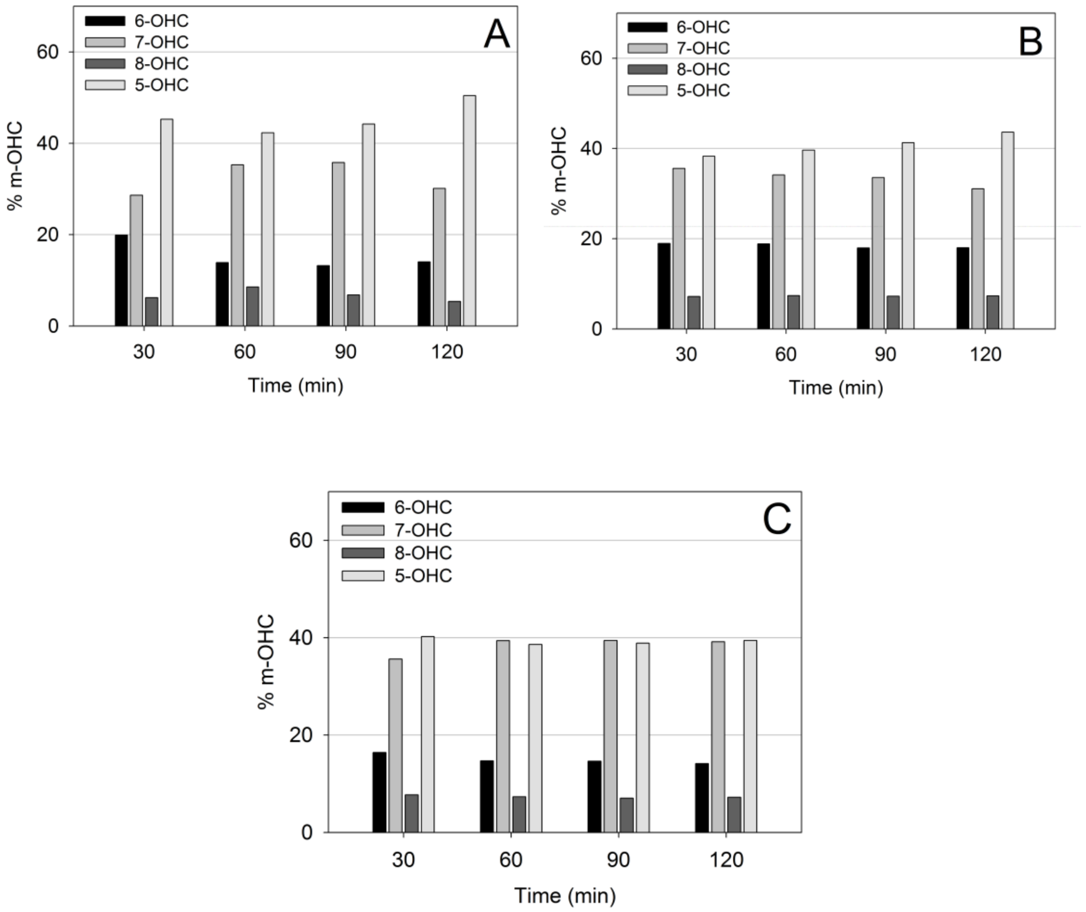

Figure S5. Percentage ratio of 5-, 6-, 7- and 8-OHC formed at selected time intervals for 100, 250 and 500 µM starting concentrations of coumarin (A,B,C, respectively).

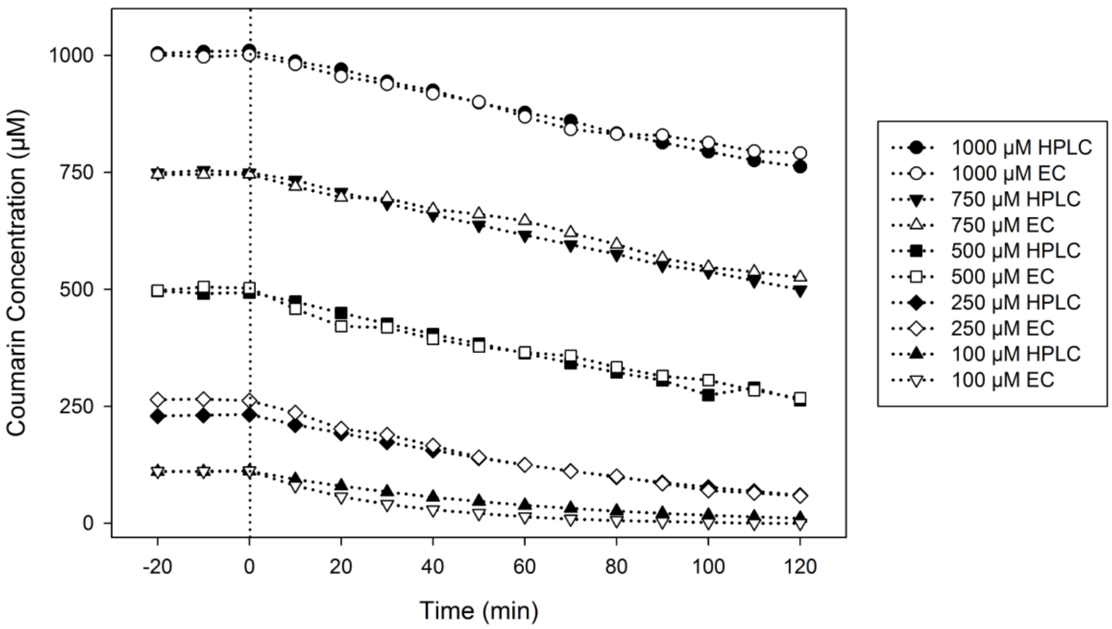

Figure S6. Concentration with irradiation time profiles for different starting concentrations of coumarin monitored by HPLC and electrochemical analysis.

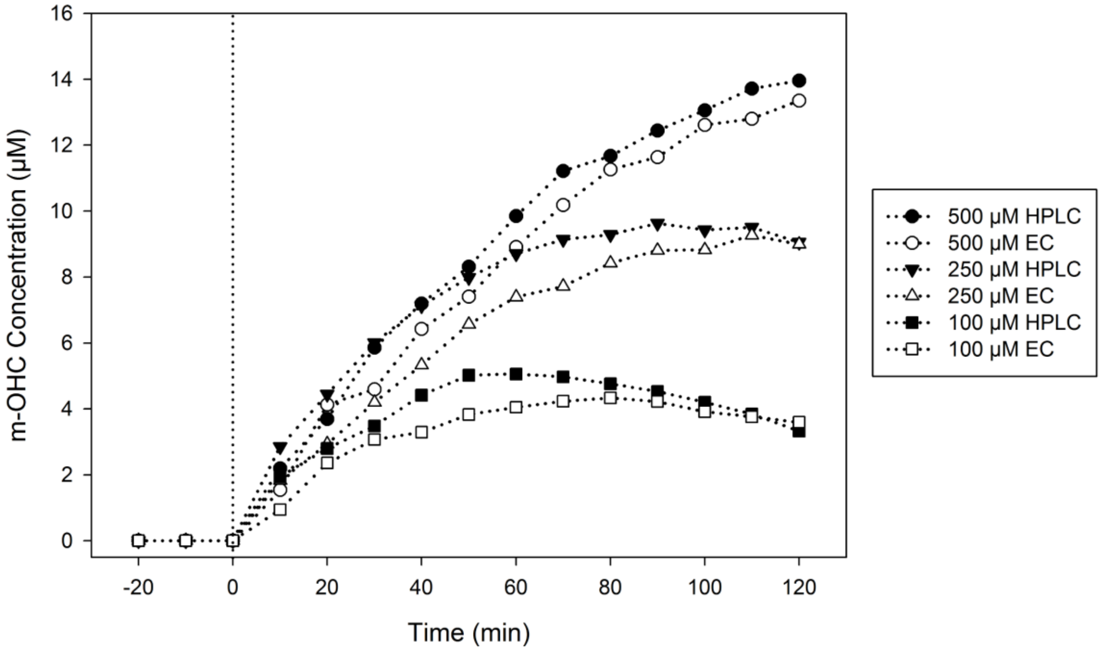

Figure S7. Comparison of HPLC and electrochemical analysis for determination of the total concentration for all m-OHC formed with irradiation time for 100, 250 and 500 µM coumarin.

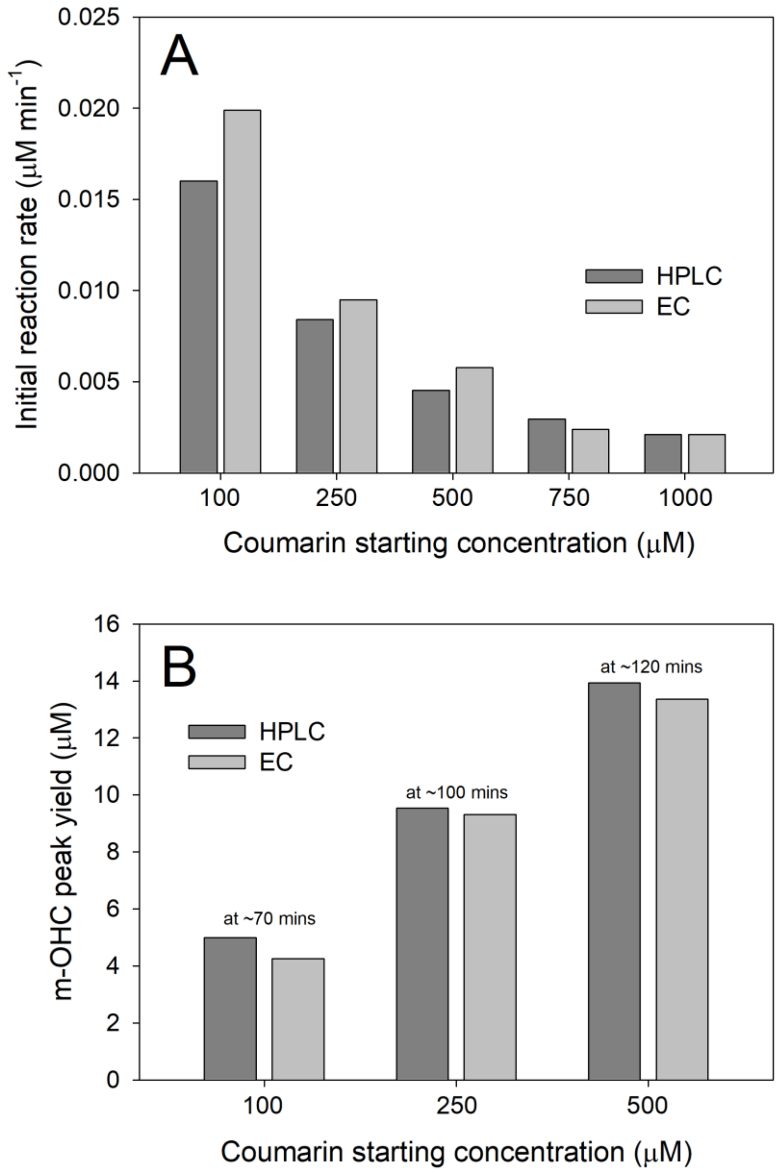

Figure S8. (A) Initial reaction rates of coumarin as its being converted to hydroxylated products using different coumarin starting concentrations by both HPLC and electrochemical monitoring. (B) The m-OHC peak yield using different coumarin starting concentrations by both detection methods. Time stated is when maximum yield occurred.
